# Risk Factors for Pancreatic Cancer: Emerging Role of Viral Hepatitis

**DOI:** 10.3390/jpm12010083

**Published:** 2022-01-10

**Authors:** Gina Gheorghe, Camelia Cristina Diaconu, Vlad Ionescu, Gabriel Constantinescu, Nicolae Bacalbasa, Simona Bungau, Mihnea-Alexandru Gaman, Madalina Stan-Ilie

**Affiliations:** 1 Department 5, Faculty of Medicine, “Carol Davila” University of Medicine and Pharmacy, 050474 Bucharest, Romania; gheorghe_gina2000@yahoo.com (G.G.); gabrielconstantinescu63@gmail.com (G.C.); mihneagaman@yahoo.com (M.-A.G.); drmadalina@gmail.com (M.S.-I.); 2 Department of Gastroenterology, Clinical Emergency Hospital of Bucharest, 105402 Bucharest, Romania; vlioax92@yahoo.com; 3 Department of Internal Medicine, Clinical Emergency Hospital of Bucharest, 105402 Bucharest, Romania; 4 Department of Visceral Surgery, “Carol Davila” University of Medicine and Pharmacy, Center of Excellence in Translational Medicine “Fundeni” Clinical Institute, 022328 Bucharest, Romania; nicolae_bacalbasa@yahoo.ro; 5 Department of Pharmacy, Faculty of Medicine and Pharmacy, University of Oradea, 410028 Oradea, Romania; simonabungau@gmail.com; 6 Department of Hematology, Center of Hematology and Bone Marrow Transplantation, Fundeni Clinical Institute, 022328 Bucharest, Romania

**Keywords:** pancreatic cancer, hepatitis B virus, hepatitis C virus, risk factors, early diagnosis

## Abstract

Pancreatic cancer is one of the most aggressive malignant neoplastic diseases. The incidence and mortality rates of this disease vary depending on geographical area, which might be explained by the different exposure to risk factors. To improve the prognosis of patients with pancreatic cancer, different approaches are needed for an earlier diagnosis. Identification of risk factors and implementation of screening strategies are essential for a better prognosis. Currently, the risk factors for pancreatic cancer fall into two broad categories, namely extrinsic and intrinsic factors. Extrinsic factors include alcohol consumption, smoking, a diet rich in saturated fats, and viral infections such as chronic infection with hepatitis B and C viruses. The pathophysiological mechanisms explaining how these hepatotropic viruses contribute to the development of pancreatic cancer are not fully elucidated. The common origin of hepatocytes and pancreatic cells in the multipotent endodermal cells, the common origin of the blood vessels and biliary ducts of the pancreas and the liver, or chronic inflammatory changes may be involved in this interaction. A careful monitoring of patients with viral liver infections may contribute to the early diagnosis of pancreatic cancer and improve the prognosis of these patients.

## 1. Introduction

Pancreatic cancer is one of the most aggressive malignant neoplasms, with a 5-year survival rate of about 10% in the United States of America (USA) [1]. The negative prognosis is due to the insidious growth and non-specific symptoms up to its advanced stages, and also to the absence of sensitive and specific methods for an early diagnosis. Approximately 80–85% of pancreatic cancer cases are diagnosed in stage IV, when the patients can no longer benefit from curative surgery [1]. In a small proportion of individuals, pancreatic cancer is diagnosed in a localized stage, when surgical treatment is possible and recommended. In this situation, the 5-year survival rate can reach up to 20% [1].

**Figure 1:**
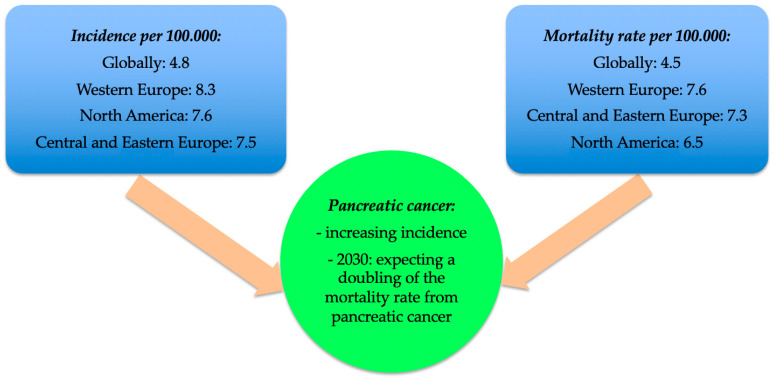
Incidence and mortality rates of pancreatic cancer [5].

In recent years, there has been an increase in the incidence of pancreatic cancer. The mortality rate attributable to this disease is expected to double by 2030 in Europe and the USA [2]. If 196,000 new cases of pancreatic cancer were reported worldwide in 1990, in 2017 this number equalled 441,000 [3]. Globally, pancreatic cancer is currently the 12th most common malignancy and the 7th leading cause of death linked to malignancy [4]. In 2018, a total of 45,918 new cases and 432,242 deaths related to pancreatic cancer were reported globally [5]. In 2019, in the USA there were 56,000 new cases of pancreatic cancer and approximately 45,000 deaths [6]. Available data suggest that in the next 20–30 years, pancreatic cancer will become the second leading cause of death attributable to cancer worldwide [1]. The worldwide incidence of pancreatic cancer is 4.8 per 100,000 individuals, with variations depending on the geographical region (e.g., 10.8 per 100,000 in Hungary and 0.35 per 100,000 in Guinea) [5]. The highest incidence rates were recorded in Western Europe (8.3 per 100,000 individuals), North America (7.6 per 100,000 individuals), and Central and Eastern Europe (7.5 per 100,000 individuals) [5]. In Romania, the incidence of pancreatic cancer is 3.3 per 100,000 individuals [7]. Regarding gender differences, the incidence of pancreatic cancer was higher among men than women (1.4 vs. 1 per 100,000 individuals) [5]. The global mortality rate was reported to be 4.5 per 100,000 individuals in 2020 [7]. The mortality rates vary with geographical region, the highest mortality rate being recorded in Western Europe (7.6 per 100,000 individuals), Central and Eastern Europe (7.3 per 100,000 individuals), and North America (6.5 per 100,000 individuals) (Figure 1) [5]. In Romania, the mortality rate of pancreatic cancer is 5.6 per 100,000 individuals and there it is the 6th leading cause of death attributable to cancer [7].

Huang et al. reported higher incidence and mortality rates of pancreatic cancer in high-income countries [5]. This suggests the possible correlation between pancreatic cancer and lifestyle factors [2]. This malignancy is associated with several risk factors that fall into two broad categories, namely intrinsic and extrinsic factors (Table 1). Extrinsic risk factors are modifiable. The higher incidence of this malignancy in developed regions can also be explained by the increasing prevalence in such areas of modifiable risk factors, such as cigarette smoking, alcohol intake, and obesity [3].

About 10% of pancreatic cancer patients have a genetic susceptibility [9]. To date, scientists have identified several genes related to the development of this malignancy, namely:

- High-penetrance genes: *BRCA2*, *STK11*, *CDKN2A*, *PALB2*;
- Low-penetrance gene: ABO blood group locus [10,11].

Regarding the hereditary risk of developing pancreatic cancer, two main categories were defined:

- Patients with genetic syndromes at risk of developing malignancies, including pancreatic cancer (e.g., Li-Fraumeni syndrome, ataxia-telangiectasia syndrome, Peutz-Jeghers syndrome, Lynch II syndrome, etc.);
- Patients at risk of familial pancreatic cancer, without a specific molecular basis [12].

In addition, the existing data in the literature suggest the possible presence of germline mutations in the genes involved in the onset of this malignancy, even in the absence of a family history of pancreatic cancer [13].

Some studies identified an association between pancreatic cancer and AB0 blood type. The blood group is determined by the presence or absence of some antigens on the surface of erythrocytes. These antigens are glycoproteins that can also be expressed on the surface of other cell types, including pancreatic cells. The relationship between pancreatic cancer and the AB0 blood group was evaluated in two large prospective studies that showed an increased risk of pancreatic cancer among individuals with non-O blood groups (A, B, AB) compared to individuals with the group O [14].

Chronic pancreatitis is characterized by a chronic inflammation of the pancreas, which can progress to pancreatic cancer [15,16]. Thus, among patients with chronic pancreatitis, a cumulative risk of progression to pancreatic cancer of 1.8% at 10 years and 4% at 20 years was reported, regardless of the type of pancreatitis [17]. A meta-analysis published in 2012 concluded a lower risk, of 1.34% at >2 years after the diagnosis of chronic pancreatitis [15]. A systematic review published in 2017 highlighted the need for a close follow-up of patients diagnosed with chronic pancreatitis to avoid delaying the identification of progression to pancreatic cancer [18]. There are also reports of the involvement of the cationic trypsinogen gene (PRSS1) in the etiopathogenesis of chronic pancreatitis and pancreatic cancer [19]. Somatic mutations of PRSS1 have been shown to play important roles in carcinogenesis [19].

The association of cystic fibrosis with some digestive cancers, including pancreatic cancer, has been demonstrated [20]. Cystic fibrosis is the most common autosomal recessive disease in Europe. It appears as a consequence of biallelic inactivating germline mutations in the cystic fibrosis transmembrane conductance regulator (CFTR) gene.

Other conditions strongly correlated with the risk of developing pancreatic cancer are pancreatic cysts, the most common types being intraductal papillary mucinous neoplasms (IPMN). Therapeutic management usually consists of carefully monitoring these patients for the early identification of possible malignant degeneration. In a surveillance strategy for IPMN, the appearance of pancreatic ductal adenocarcinoma was reported in 2–9% of patients, independent of the evolution of IPMN. These results suggest the existence of genetic defects in the pancreatic tissue [21].

The relationship between pancreatic cancer and diabetes mellitus is bidirectional. Thus, approximately 1% of patients over the age of 50 years, newly diagnosed with metabolic disorder, associate pancreatic cancer as a trigger for their diabetes [22]. In addition, patients diagnosed with diabetes mellitus less than one year previously were found to have a higher risk of developing pancreatic cancer compared to those with long-term diabetes (5.4 vs. 1.5) [22]. However, more and more data support the fact that abnormal glucose metabolism, insulin resistance, and hyperinsulinemia are rather etiological factors for the development of this malignancy [23,24]. A meta-analysis of 88 studies reported a 1.97-fold increase in the risk of pancreatic adenocarcinoma among patients diagnosed with diabetes mellitus [25]. There are also data suggesting that patients with diabetes and pancreatic cancer have a worse prognosis compared to those who do not associate diabetes [26].

A study conducted in the United States on a group of 14,672,409 individuals identified an association between obesity and a variety of cancers depending on the patient’s age. In the 25–49 years age group, the authors found an association between obesity and multiple myeloma, pancreatic, colorectal, kidney, gallbladder, and uterine corpus cancer [27]. Lipomatous infiltration of the pancreas was associated with the development of pancreatic adenocarcinoma precursor lesions, namely pancreatic intraepithelial neoplasia [28].

Michaud et al. reported a significantly higher relative risk of developing pancreatic cancer among individuals with a body mass index (BMI) > 30 kg/m^2^ compared to individuals with a BMI < 23 kg/m^2^ (1.72 vs. 1.19) [29]. In addition, other studies suggest that overweight or obese individuals develop pancreatic cancer at a younger age compared to people of normal weight. In addition, their survival rates are lower compared to individuals with the same diagnosis, but normal weight [30]. Regarding the relationship between the diet and the risk of pancreatic cancer, the data are contradictory. On one hand, a series of studies have shown a directly proportional relationship between diets high in saturated fats and/or meat (particularly processed/smoked meat) and the risk of developing this neoplasm. On the other hand, a protective effect is played by diets rich in fresh fruit and vegetables [31]. However, this information has not been validated in prospective studies [32]. Another important observation is the relationship between low levels of lycopene (carotenoid present in fruit) and selenium and the subsequent development of pancreatic cancer [8,33]. However, the role of dietary supplementation with these nutrients in reducing the risk of developing pancreatic cancer is unclear [34].

Another very important risk factor for the development of pancreatic cancer is smoking. According to the available data, the risk of developing pancreatic cancer is two times higher among smokers compared to non-smokers [35,36]. One study estimates that approximately 11–32% of pancreatic cancer cases are associated with tobacco consumption [35,37]. This risk increases with the number of packs per year, and it is higher in individuals who associate homozygous deletions of the gene for the enzyme that metabolizes glutathione S-transferase theta 1 (GSTT1) [38]. Smoking cessation can lead to a significant reduction in the risk of developing pancreatic cancer. It has been shown that after two years of smoking cessation, the risk of pancreatic cancer decreases by about 48%, and after 10–15 years the risk reaches a level compared to that seen in non-smokers [39]. Moreover, data from the USA support a reduction in the number of deaths attributed to these neoplasms of approximately 25% by stopping tobacco use [39].

The relationship between pancreatic cancer and alcohol consumption is dependent on both the type of drink and the amount consumed. Thus, a case-control study conducted in 2010 identified an association between the development of pancreatic cancer and the consumption of 60 g of liquor per day, while the consumption of wine and beer has not been shown to be associated with this neoplasm [40,41]. It is noteworthy that only excessive alcohol consumption, and not reduced or moderate drinking, is a risk factor for pancreatic cancer [8].

The detailed analysis of some studies that reported the correlation between these risk factors and pancreatic cancer highlighted the association of smoking with alcohol or coffee consumption, without being able to exclude a weak or false positive correlation between the last two factors and the development of this malignancy [41,42,43].

Another possible extrinsic risk factor associated with pancreatic cancer is the prolonged use of nonsteroidal anti-inflammatory drugs. A study that included 88,378 women without cancer over 18 years reported a possible elevated risk of pancreatic cancer among the individuals who used acetylsalicylic acid regularly (>14 tablets of acetylsalicylic acid per week) [44]. However, another study that included 987,590 adults from the USA did not confirm this association. Thus, there was no evidence of increased risk of pancreatic cancer even among individuals who used acetylsalicylic acid ≥30 days/month for more than 20 years [45].

The presence of hepatitis B and C and the infection with *Helicobacter pylori* correlate positively with the risk of pancreatic cancer [8]. There are also data in the literature that suggest an association between the non-O blood group and colonization with *Helicobacter pylori* [14]. Anti-*Helicobacter pylori* antibodies were identified only in patients with non-O blood groups. One theory suspects that the variation of the binding capacity of *Helicobacter pylori* in the gastro-intestinal tract depends on the terminal binding antigen in mucins at this level. The type of these terminal antigens is dependent on the blood group [46,47]. It is also known that this bacteria is involved in the pathogenesis of other neoplasms, such as gastric carcinoma and lymphoma [48].

Other studies suggest a possible relationship between various diseases of the oral cavity, such as periodontitis, and increased risk of pancreatic cancer [49,50]. Farrell et al. suggest the involvement of microorganisms of the oral flora, such as *Neisseria elongate* and *Streptococcus mitis*, in the development of pancreatic diseases, including malignant tumors [51].

Some studies identify viral infections among the risk factors for pancreatic cancer. Thus, a meta-analysis of observational studies conducted in 2013 showed an increased risk of pancreatic cancer among patients with chronic liver infections with the hepatitis B virus (HBV) and the hepatitis C virus (HCV) [52]. HBV and HCV are hepatotropic viral agents with oncogenic properties and they possess an ability to integrate their viral DNA/RNA into the genome of infected cells [52,53].

## 2. Viral Hepatitis: Virology and Epidemiology

Viral hepatitis is a worldwide public health problem because of the large number of affected individuals and the morbidity and mortality rates associated with these infectious disorders. Among viruses responsible for hepatitis, the most important are the hepatitis A virus (HAV), hepatitis B virus (HBV), hepatitis C virus (HCV), hepatitis D (delta) virus (HDV), and hepatitis E virus (HEV) [53]. Of these, the only one that does not lead to chronic hepatitis is HAV, while HBV, HCV, HDV, and occasionally HEV may be responsible for long-term forms of viral liver infection [53]. Worldwide, it is estimated that approximately 2 billion people have liver alterations secondary to an active or inactive form of HBV infection, while 257 million people are chronic carriers of HBV. There are approximately 71 million chronic carriers of HCV [53,54]. Globally, the prevalence of individuals positive for hepatitis B surface antigen (HBsAg) is 3.5%, but the rate varies depending on the geography [54]. Thus, the prevalence of chronic hepatitis B is between 1.2–2.6% in Europe, 0.4–1.6% in the USA, 4.6–8.6% in Africa, 1.5–4.0% in the Eastern Mediterranean, and 5.1–7.6% in the Western Pacific [54]. The prevalence of chronic hepatitis C also varies by geographical area [55]. The most affected regions are the Eastern Mediterranean, with a prevalence of 2.3%, and Europe, with a prevalence of 1.5%. It is noteworthy that approximately 23% of new HCV infections and 33% of HCV-attributed mortality correlate with the use of intravenous drugs [55]. In terms of mortality, a report published in 2017 estimated that in 2015 there were 1.4 million deaths as a consequence of viral hepatitis [56]. In more than 90% of cases, these deaths resulted because of either liver cirrhosis or hepatocellular carcinoma (HCC), conditions related to HBV or HCV infection [56].

HBV is a DNA virus, belonging to the *Orthohepadnavirus* genus, and is classified into 10 genotypes, A–J [56]. This virus can be transmitted sexually, by blood, and vertically, from the mother to the fetus [56]. HBV genotypes have several peculiarities in terms of geographical distribution, route of transmission, and organ damage [57].

HDV is a virusoid (defective virus) that can only replicate in the presence of HBV. HDV can be transmitted simultaneously with HBV (coinfection) or an HDV infection can later overlap with a chronic HBV infection (superinfection). About 5–10% of patients infected with HBV associate an HDV infection. This conglomerate of viral infections is more common among people who use intravenous drugs [53].

HCV is an RNA virus that is part of the *Flaviviridae* family. It is divided into eight genotypes (1–8) [53]. The only natural reservoir of this virus are humans and interpersonal transmission is mainly through blood products [53]. Perinatal transmission occurs in 3–10% of children born to HCV-infected mothers [58]. An increased risk of sexual transmission has also been reported among men who have sex with men and are infected with the human immunodeficiency virus (HIV) [59]. Among the possible outcomes of the HCV infection, about 15% of cases show spontaneous clearance of the virus, less than 15% of cases develop into acute forms of viral hepatitis, and the rest develop chronic forms of viral hepatitis [53]. About 30 years after the infectious encounter, up to a third of these patients progress to liver cirrhosis [53]. The risk factors for liver cirrhosis development in HCV-infected patients are male sex, HBV co-infection, HIV infection, schistosomiasis, obesity, insulin resistance, and chronic alcohol consumption [53,60,61]. Patients with liver cirrhosis secondary to HCV infection have an annual risk of 1–5% of developing hepatocellular carcinoma and a 3–6% risk of liver decompensation [53].

## 3. Hepatotropic Viruses and Pancreatic Cancer-Pathophysiological Links

HBV and HCV are hepatotropic viruses with oncogenic properties. These viruses can induce persistent liver injury, with subsequent progression to cirrhosis and hepatocellular carcinoma [62,63]. There are also data suggesting the involvement of these viruses in the development of other neoplasms, such as pancreatic cancer, intrahepatic and extrahepatic duct bile carcinoma, gastric cancer, oesophageal cancer, certain forms of non-Hodgkin’s lymphoma, and leukemia [64,65,66,67].

Anatomically, the pancreas is situated in the proximity of the liver. The common origin of the blood vessels and bile ducts of these organs makes possible the microorganisms’ migration [52]. Antigens and replicative sequences of these two viruses have also been identified in extrahepatic tissues (e.g., the pancreas, the kidneys, and the skin) [68,69].

Hoefs et al. provided the first evidence of pancreatic HBV replication in 1980 by detecting HBsAg in the pancreatic juice of individuals with confirmed HBV infection [70]. Subsequently, the presence of HBsAg and HBV core antigen in the cytoplasm of pancreatic acinar cells was demonstrated [71], together with the integration of HBV-DNA in the pancreatic tissue and in liver metastases of pancreatic origin in HBV-infected patients [72]. Recurrent HBV hepatitis after liver transplant possibly supports the existence of extrahepatic reservoirs of this virus [68,73]. HCV antigens have also been identified in pancreatic acinar cells, demonstrating the possible replication of this virus in the pancreas [74].

Another observation that indicates the involvement of these viruses in the pathogenesis of pancreatic cancer is the impairment of exocrine pancreatic function in patients with chronic hepatitis [75]. Thus, some studies have shown an increase in serum and urinary levels of pancreatic enzymes in patients with chronic HBV and HCV hepatitis [76,77].

Some studies support acute pancreatitis as an extrahepatic manifestation or complication that can occur in the evolution of certain forms of acute, fulminant, or chronic hepatitis [78,79]. A study that analysed a group of 476 patients with pancreatic adenocarcinoma, with the diagnosis confirmed by histopathological examination, and a control group of 876 individuals without pancreatic cancer concluded that the previous exposure to HBV may be associated with pancreatic cancer [75]. The same study found an association between occult HBV infection (positivity for antibodies against the HBV core antigen, negativity for the HBV surface antigen, and negativity for antibodies against the HBsAg) and pancreatic cancer [75]. This raises the hypothesis of HBV reactivation among patients receiving chemotherapy [75]. When the use of chemotherapeutic agents is ceased, these subjects may develop liver failure as the immune system recovers and attempts to eradicate the infected liver cells [75].

The presence of these viruses in pancreatic tissue induces chronic inflammatory changes, with the possibility of progression to metaplasia and subsequently to malignant transformation [80]. The inflammatory microenvironment leads to an accumulation of growth factors and cytokines, with secondary alterations of driver genes and stimulation of cancer cells’ growth and proliferation [81].

There are also data that support the etiological relationship between HBV, HCV, and acute pancreatitis. The pathophysiological mechanisms incriminated include viral replication in the pancreas, the immune response, or the direct cytotoxic effect on pancreatic acinar cells [82]. Some authors also suggest edema of the ampulla of Vater and secondary obstructions of the drainage of pancreatic juice as a possible pathophysiological mechanism [82,83]. Another cause of acute pancreatitis secondary to hepatotropic virus infection may be represented by the disruption of the pancreatic flow [82,84]. On the other hand, patients with acute non-vascular pancreatitis appear to develop rapid disturbances of the pancreatic microcirculation [84]. A study that included patients with acute pancreatitis secondary to hepatotropic virus infection reports the possibility of their progression to chronic pancreatitis, a major risk factor for pancreatic cancer, within 6–36 months [85].

Another hypothesis that supports the possibility of these viruses’ involvement in the occurrence of pancreatic cancer is the common origin of hepatocytes and pancreatic cells in the multipotent endodermal cells, with the possibility of transformation when cultured under specific conditions [86,87,88]. Under these conditions, the pancreas can be a reservoir for HBV/HCV, but it can also ensure optimal conditions for the replication of these viruses.

A study published in 2013 supports the association between HCV infection and other risk factors for pancreatic cancer, such as smoking, alcohol consumption, chronic pancreatitis, and diabetes, with the possibility of confusion regarding the relationship between pancreatic cancer and HCV infection [81].

If in the case of pancreatic cancer, the pathophysiological mechanism by which these hepatotropic viruses contribute to the development of malignancy is still unclear, in the case of hepatocellular carcinoma there is some clear evidence. HBV-DNA integration at the cellular level contributes to chromosomal instability and secondary hepatocellular carcinoma development [89]. The oncogenic role of hepatitis B viral proteins (HBx proteins) has also been documented. These proteins can lead to the activation of both HBV genes and genes that are normally present at the cellular level. This results in an alteration of gene expression, with a risk of developing HCC [90]. Inflammatory and necrotic processes induced by viral replication in the liver also play a role in the development of HCC [81]. One study showed the expression of HBx proteins in tumor tissue harvested from pancreatic or gastric tumors [91]. This led to the hypothesis that chronic HBV infection induces lesions of gastric and pancreatic epithelial cells, with the possibility of further evolution to malignancy [92,93].

A meta-analysis, published in 2013, which assessed the association between viral hepatitis and pancreatic cancer, found an increased risk of pancreatic cancer by 20% in HBV-infected patients and by 23% in HCV-infected patients compared to the general population [52]. This meta-analysis highlights the importance of the chronicity of the infection and the fact that active replication of HBV does not increase the risk of pancreatic cancer. Furthermore, seroconversion from HBsAg to anti-HBs decreases the risk of pancreatic cancer [52].

Several prospective studies conducted in the past year also investigated the potential interplay of viral hepatitis and pancreatic cancer. For example, Liu et al. (2021) prospectively evaluated 93,402 Chinese subjects with chronic hepatitis B for a period of approximately 13 years. During this time frame, the researchers recorded 1791 cases of gastrointestinal malignancies in the study group and depicted a direct association between hepatitis B and the onset of pancreatic cancer [hazard ratio (HR) = 1.86, 95% confidence interval (CI): 1.10–3.99]. Overall, subjects who displayed positivity for HBsAg had an elevated risk of being diagnosed with a gastrointestinal malignancy (HR = 5.59, 95% CI: 4.84–6.45) versus HBsAg-negative individuals. Although the risk of cancer was more notable for HCC (HR = 21.56, 95% CI: 17.32–26.85), and gallbladder or extrahepatic bile duct cancer (HR = 14.89, 95% CI: 10.36–21.41), the risk of pancreatic (HR = 1.86, 95% CI: 1.10–3.99) and colorectal cancer (HR = 1.75, 95% CI: 1.15–2.96) development in HBs-Ag-positive individuals was not negligible [94]. Moreover, Lam et al. (2021) evaluated the risk of cancer development in subjects diagnosed with hepatitis C in the USA. In their cohort study of 2451 HCV-positive and 173,548 HCV-negative individuals, the investigators noted an elevated risk of HCC, pancreatic cancer, and haematological malignancies [adjusted incidence rate ratio (aIRR) = 31.4, 95% CI: 28.9–34.0, aIRR = 2.0, 95% CI = 1.6–2.5 and aIRR = 1.3, 95% CI = 1.1–1.5, respectively]. The use of direct-acting antivirals decreased the risk of liver and hematological malignancies. However, it did not impact the risk of pancreatic cancer, which can give rise to the hypothesis that HCV can contribute to the onset of this neoplasm [95]. Furthermore, anti-HBs antibodies were found to be protective against the development of pancreatic cancer [adjusted odds ratio (aOR) = 0.58, 95% CI: 0.42–0.82] in a Chinese case-control study (cases: 4748 cancer subjects; controls: 57,499 subjects), revealing that vaccination against this HBV could reduce the risk of malignancies of the pancreas [96]. However, we must point out that the risk factors for pancreatic cancer, including exposure to viral agents, are different around the globe, in addition to other non-modifiable risk factors that could influence the development and/or prognosis of this malignancy (e.g., genetics, ethnicity/race, and others) [97]. Overall, surveillance and screening programs conducted because of international efforts have paved the road toward the elimination of viral hepatitis, which remains a public health threat, particularly in low-/middle-income countries where cancer-screening programs, including those dedicated to pancreatic cancer, are deficient [97,98,99]. As pancreatic cancer remains the malignancy with the most elevated case fatality rate and whose age-standardized death rate increased by 24% among 29 cancer groups based on the assessment of the 1990–2019 trends in the global cancer burden, screening programs for the early detection of this disorder are of paramount importance for the near future [99]. Thus, we strongly believe that the management of modifiable risk factors, including the potential role of the exposure to viral agents such as HCV and HBV, can have a positive impact on (but is not restricted to) pancreatic cancer care and can reduce the financial and human costs of this malignancy.

**Figure 2:**
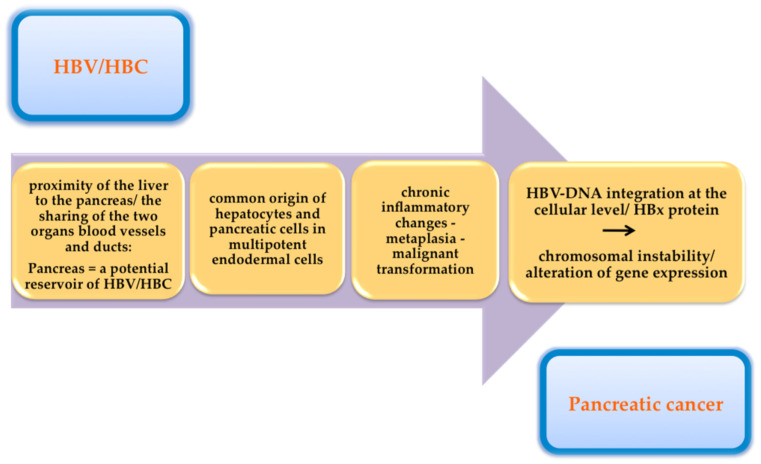
The pathophysiological mechanisms by which hepatotropic viruses may contribute to the development of pancreatic cancer.

Figure 2 summarizes the pathophysiological mechanisms by which hepatotropic viruses may contribute to the development of pancreatic cancer.

## 4. Conclusions

Pancreatic cancer is a disorder with a poor prognosis mainly because of its late diagnosis. The symptomatology remains nonspecific until its advanced stages, and the retroperitoneal location of the pancreas makes imaging diagnosis more difficult. To improve the prognosis of patients with pancreatic cancer, different approaches are needed for an earlier diagnosis. Viral infections with HBV and HCV are listed among the risk factors involved in the development of pancreatic cancer. The pathophysiological mechanisms by which these hepatotropic viruses contribute to the development of pancreatic cancer are not fully elucidated. Among the possible mechanisms are the anatomical proximity of the two organs, the common origin of the blood vessels and bile ducts of these organs, and the possibility of microorganism migration, the common origin of hepatocytes, and pancreatic cells in multipotent endodermal cells, with the risk of transformation when cultured under specific conditions (the pancreas can be a reservoir for HBV/HCV), and chronic inflammatory changes, with a possible progression to metaplasia and subsequently to malignant transformation, HBV-DNA integration at the cellular level with chromosomal instability, and alteration of gene expression. Future studies are needed to evaluate this relationship and to clarify the pathophysiological mechanisms. Also, there is a need for prospective studies to evaluate the most appropriate surveillance strategy for patients with chronic viral hepatitis regarding the risk of pancreatic cancer, in terms of performance and cost-efficiency. The virus clearance by antiviral treatment may have a role in decreasing the risk of pancreatic cancer.

## Figures and Tables

**Table 1:** Risk factors for pancreatic cancer [1,8].

Intrinsic Risk Factors	Extrinsic Risk Factors
Hereditary	Diet
AB0 blood group	Obesity
Chronic pancreatitis	Tobacco
Cystic fibrosis	Coffee and alcohol consumption
Pancreatic cysts	*Helicobacter pylori* infection
Diabetes mellitus and insulin resistance	Infection with hepatitis B virus (HBV) and hepatitis C virus (HCV)
